# The effect of individual drilling sleeves on the precision of coronectomy tooth sections. An in vitro 3D-printed jaw model experiment

**DOI:** 10.1007/s00784-023-05289-4

**Published:** 2023-10-03

**Authors:** Ana Pacheco, Balázs Soós, Edina Lempel, Imre Simon, Péter Maróti, Stephan Christian Möhlhenrich, József Szalma

**Affiliations:** 1 Department Oral and Maxillofacial Surgery, Medical School, University of Pécs, 1. Tüzér St, 7623 Pécs, Hungary; 2 Department of Conservative Dentistry and Periodontology, Medical School, University of Pécs, 1. Tüzér St, 7623 Pécs, Hungary; 3 3D Printing and Visualization Centre, University of Pécs, 2. Boszorkany St, 7624 Pécs, Hungary; 4 Medical Skills Education and Innovation Centre, Medical School, University of Pecs, 12. Szigeti St, 7624 Pécs, Hungary; 5 Department of Orthodontics, University of Witten/Herdecke, Alfred-Herrhausen Str. 45, 58455 Witten, Germany

**Keywords:** Third molar, Coronectomy, 3D-printing, Drill sleeve, Lingual nerve damage

## Abstract

**Objectives:**

The aim of this in vitro study was to evaluate the effect of a 3D-printed drill sleeve (DS) on the precision and duration of coronectomy sections.

**Materials and methods:**

Thirty-six trainees and oral surgeons performed 72 coronectomy cuts in a 3D-printed, entirely symmetric mandible model. Coronectomy was performed freehand (FH) on one side and with a DS on the other side. The occurrence of “too superficial” (≥ 4 mm unprepared lingual tooth tissue) and “too deep” (drilling ≥ 1 mm deeper as tooth contour) cuts and sectioning times were registered.

**Results:**

In 7 cases, the sections were “too deep” with FH, while none with DS (OR: 18.56; 95%CI: 1.02–338.5; p = 0.048). The deviation between virtually planned and real cut depths was significantly greater in the FH group (1.91 ± 1.62 mm) than in DS group (1.21 ± 0.72 mm) (p < 0.001). A total of 18 “too superficial” buccolingual sections occurred with FH, while 8 cases with DS (OR: 3.50; 95%CI: 1.26–9.72; p = 0.016). Suboptimal sections did not correlate with experience (p = 0.983; p = 0.697). Shortest, suboptimal drillings were most frequently seen distolingually (OR: 6.76; 95% CI: 1.57–29.07; p = 0.01). In the inexperienced group, sectioning time was significantly longer with FH (158.95 ± 125.61 s vs. 106.92 ± 100.79 s; p = 0.038).

**Conclusions:**

The DS effectively reduced tooth sectioning times by less experienced colleagues. Independently from the level of experience, the use of DS obviated the need for any preparation outside the lingual tooth contour and significantly decreased the occurrence of “too superficial” cuts, leaving thinner unprepared residual tooth tissue lingually.

**Clinical relevance:**

Coronectomy sections may result in lingual hard and soft tissue injury with the possibility of damaging the lingual nerve. The precision of the buccolingual depth-control can be improved, while surgical time can be reduced when applying a drilling sleeve.

**Supplementary Information:**

The online version contains supplementary material available at 10.1007/s00784-023-05289-4.

## Introduction

Third molar surgery is definitely one of the most frequently performed interventions in oral surgery. Major complications include mandible fractures, inferior alveolar nerve (IAN) and lingual nerve (LN) injuries, tooth dislocations in facial spaces, and severe postoperative inflammations. A cohort study identified third molar-related operations as the most frequent reason (45%) for iatrogenic nerve injury [1]. Partial odontectomy [2], extraction with orthodontic forces [3], and pericoronal ostectomy [4] of third molars were introduced to reduce postoperative neurosensory disturbances of the IAN.

To decrease the chance of IAN injury and also for partial tooth removal, coronectomy was introduced in 1984 by Ecuyer and Debien and in 1989 by Knutsson et al. [5, 6]. Although there is no generally accepted guideline for coronectomy, the basis of the method is the removal of the coronal part after sectioning the tooth with deliberate retention of the roots, thereby avoiding potential damage of the IAN [7]. Besides of the advantages of the technique, possible intraoperative and postoperative complications have to be mentioned. Among intraoperative risks, mobilization of tooth roots shows the highest occurrence, ranging from 3 to 9%, based on Patel et al. [8]. However, a higher probability (38.5%) was also described in the literature [9]. Damage of the IAN, lingual nerve (0–2%) and lingual soft tissues should also be highlighted, while damage to the second molar can also occur. Similar to total tooth extraction, the most common postoperative complications are pain (1.1–41.9%), alveolitis (2–12%), facial swelling (4.6%), pulpitis (0.9%) and other additional infections (1–9.5%) [10]. The conditions listed above are classified as short-term postoperative consequences. Root canal treatment at the time of coronectomy cannot be recommended [11]. Additionally, it is important to mention root migration (2–85.3%) and eruption, which are considered long-term postoperative consequences and can lead to subsequent inflammation, even after 8–10 years [7, 12]. Long-term follow-up of patients is an additional challenge due to the possibility of these late complications.

Approximately 32% of malpractice claims are because of third molar surgery–related IAN or lingual nerve damage [13], so it is obvious that some authors emphasize the importance of offering coronectomy [14].

According to Yeung et al., in 2019, 79 publications were found regarding coronectomy, with an average of 9.7 citations per article [15]. In 2019, the five countries showing the highest publication rate were the United Kingdom (25.3%), the United States (12.7%), Italy (11.4%), China (6.3%) and Turkey (6.3%) [15]. Currently, searching for the keyword “coronectomy” in the PubMed database yields 139 human-related results, most of which were published after 2009 (90.6%). The type of published articles is most often review (14.4%), case report (9.4%), comparative study (9.4%), systematic review (8.6%), clinical study (7.9%), clinical trial (7.9%), controlled clinical trial (6.5%), randomized controlled trial (5%) and meta-analysis (4.3%). In terms of the latest 50 relevant announcements, the most frequent publishing countries are China (22%), the UK (12%), the USA (8%) and Italy (8%), but the number of publications from Brazil, Denmark, Hungary, India, Japan, the Netherlands, Saudi Arabia, South Korea, Switzerland, Taiwan and Turkey is also worth mentioning. A publication stated that coronectomy is very well accepted in the UK and the USA [16], while another one stated that ~ 48% of Swiss colleagues use coronectomy [17]. The same study revealed that coronectomy’s acceptance among colleagues graduating after 2005 is significantly higher. China’s role in the spread of technology and new technical modifications should also be highlighted, including the combination of coronectomy with miniscrew traction described by Zhao et al. [18].

Even though the technique’s international appreciation is changeful, numerous literature data support the effectiveness of coronectomy in reducing the risk of IAN injury [9, 19–23]. In a recent paper, the following statement was concluded: “Coronectomy is an established and effective alternative to extraction in cases with a high risk of injury to the IAN” [24].

Based on these, while the risk of temporary IAN injury in traditional extraction of high-risk lower third molar is about 5–19%, the incidence of injuries of the IAN caused by coronectomy did not exceed 5.7% [20]. In addition, based on the literature, the risk of permanent nerve damage is negligible [9, 19–23].

Regarding coronectomy tooth sections, numerous investigations can be found in the literature [20, 22, 25, 26]. The inclination of the horizontal buccolingual cut [22, 27], the role of a possible adjacent vertical buccolingual cut of the crown [25], making more sections to weaken the crown [20], the effect of different instrumentation on sectioning time, cutting surface quality, and intra-osseal heat have been investigated earlier [26].

Proper tooth sectioning is crucial in coronectomy because root/s can be mobilized when decoronating-attempt (crown fracture) is made after a superficial, inadequate cut. When the root/s of the third molar are mobilized, they must be removed; thus, an unsuccessful coronectomy has to be modified to tooth removal, meaning the increased risk for IAN injury [9, 22]. In contrast, a too deep or an uncontrolled buccolingual cut may harm lingual alveolar bone or lingual soft tissues, or both, endangering the lingual nerve [28]. Additionally, even in pronounced distal or horizontal tooth angulations, the IAN can be injured when inferior alveolar canal is in close contact with the tooth at the site of section.

Furthermore, the experience of the surgeon was found to be a significant factor in the incidence of intra- and postoperative complications of third molar removal [29]. Four times higher complication rate was observed in the case of trainees than that in the case of experienced surgeons in third molar removals. Interestingly, this difference was observed both in partially erupted and full bony impacted cases [29].

To reduce drilling-related complications during third molar surgery, several techniques and proposals have been introduced. These include dynamic image navigation [30], 3D-printed drilling sleeves [31], fully guided tooth bud microwave ablation [32], or digital tooth sectioning guide systems [33]. The 3D-printed drilling sleeve for coronectomy was invented and published in 2019 by our research team as a cost-effective and easily used tool, aiming to decrease complication rate and to increase self-confidence not only for practicing oral surgeons but also for preclinical education during simulation-based training [31]. Although the device has been thoroughly tested clinically in the meantime, clear and objective evidence-based benefits had to be validated among colleagues with different levels of experience.

The aim of this 3D-printed jaw model experiment was to investigate the effectiveness of a 3D-printed drilling sleeve on tooth sectioning time and buccolingual depth-control of coronectomy procedures during drilling by oral and maxillofacial trainees and surgeons with different levels of experience.

## Materials and methods

This 3D-printed jaw model experiment was conducted in the oral surgery teaching unit of the Department of Oral and Maxillofacial Surgery, Medical School, University of Pécs (Pécs, Hungary). To perform imaging and to use diagnostic and clinical treatment data of patients regarding coronectomies for study purposes, ethical approval (IRB: 7920/PTE/2019) was obtained.

### Designing the jaw-model

The cone-beam computed tomography (CBCT) of a coronectomy patient who had been treated earlier was used for the segmentation and fabrication of a 3D-printed lower jaw model. The reason for which this case was selected was that there was a slight lingual inclination of the tooth, which resulted in a moderately difficult lingual visual control clinically during coronectomy.

**Fig. 1:**
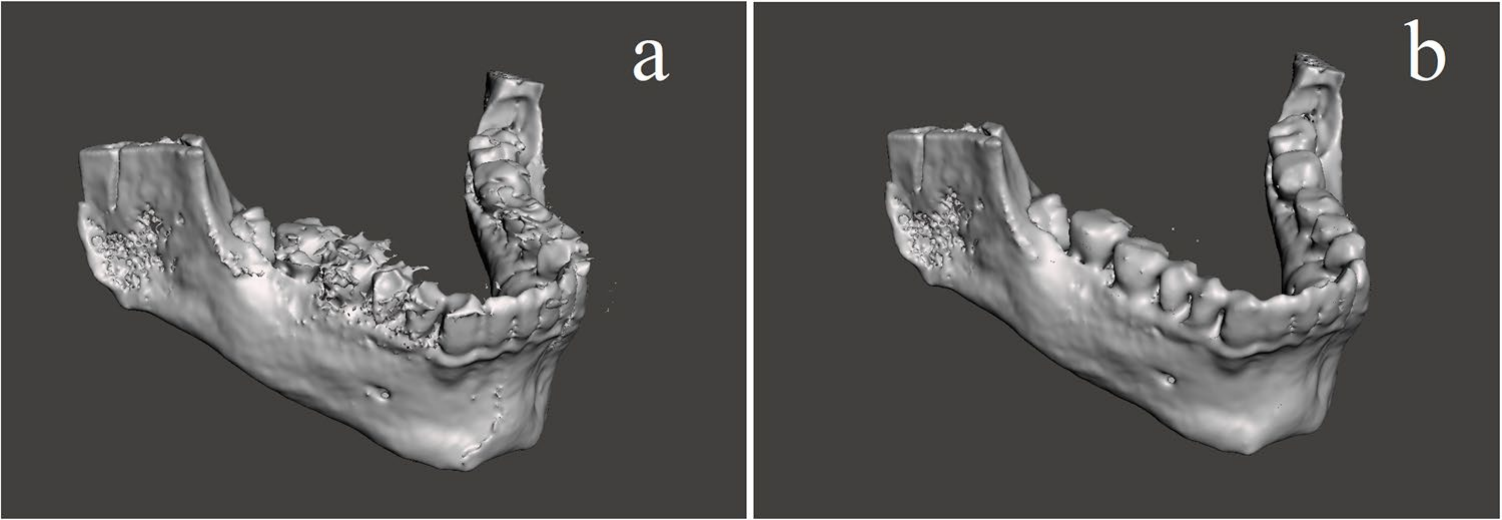
**a** The.stl file exported from the 3D Slicer (version 5.0.3). **b** The.stl file after the smoothing algorithm was applied in Meshmixer (Autodesk, San Rafael, CA, USA)

For the CBCT, a GXDP-800 3D unit (KAVO- Gendex, Charlotte, NC, USA) [90 kVp; 3.2–10 mA/6.1–8.5 s; FOV 61 × 78 mm or 78 × 150 mm; focal spot 0.5 mm; scan time 10–20 s; slice thickness 0.5 mm; voxel size 0.2 mm] was used. Digital imaging and communications in medicine (DICOM) data from the CBCT scans were then imported to the medical imaging software 3D Slicer (version 5.0.3, free and open-source medical image computing platform for biomedical research) for segmentation to reconstruct 3D volumetric data and exported as STereoLithography (.stl) file (Fig. 1 a). The.stl files were then imported to the 3D processing software Meshmixer (Autodesk, San Rafael, CA, USA) (Fig. 1 b). To retain the anatomical features of the scanned mandibular region, only the minimum smoothing algorithm was applied to eliminate noise on the teeth and bony structures.

**Fig. 2:**
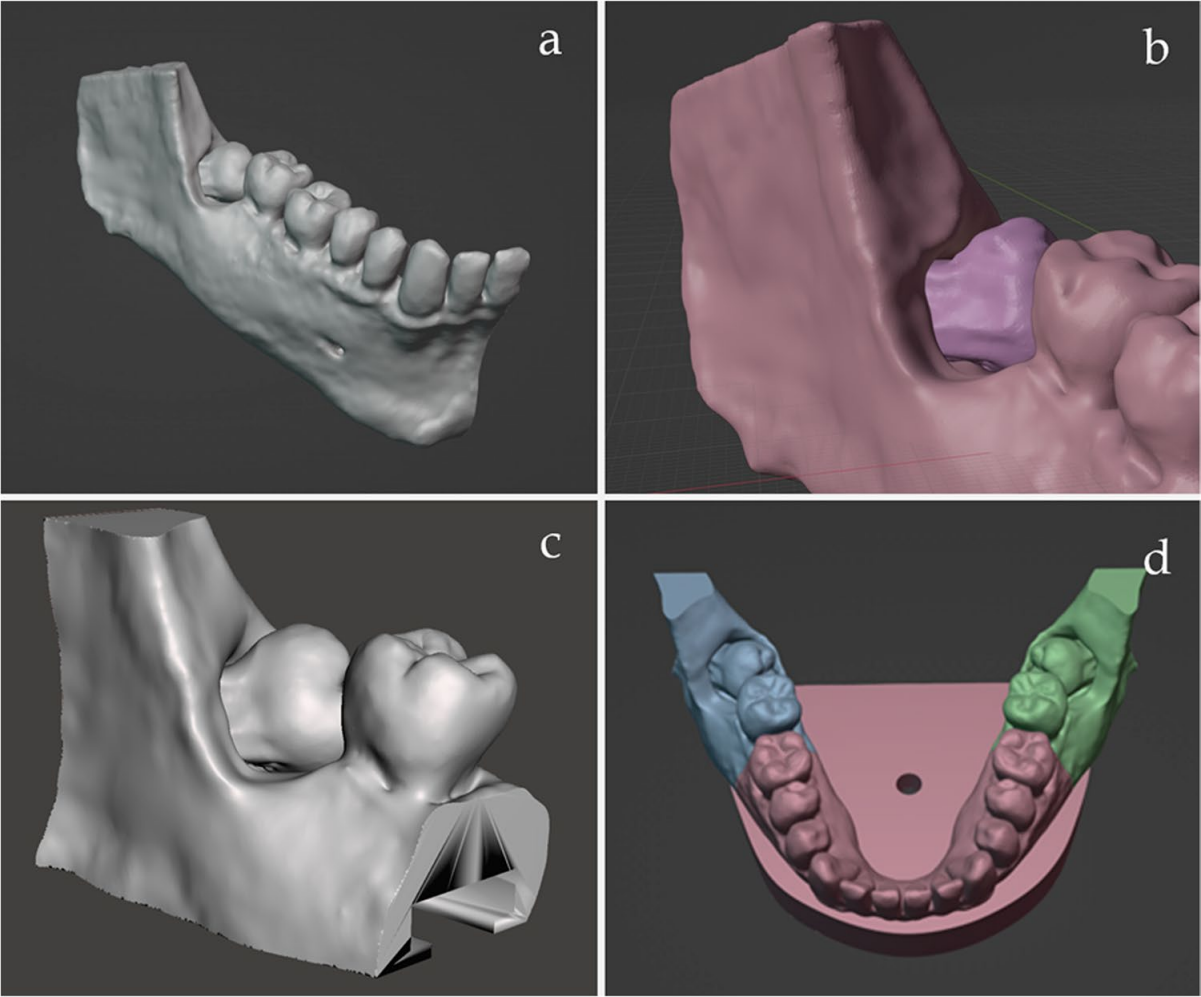
**a** The right side of the model with slightly redefined teeth **b**) The bone crest that was removed **c**) The distal molar areas were made changeable **d**) The right side of the model was mirrored, and the base of the model was designed

Then, the.stl file was imported to the Blender software (version 3.2, open source, released under the GNU General Public License), where modifications were made. The model was cut in the middle in object mode by creating a cube and using the Boolean function. On the right side of the model, the shape and the occlusal surfaces of the teeth were slightly redefined in sculpt mode. (Fig. 2 a). Buccally there was a bone crest on the original scanned and smoothed jaw image obtained from the patient, that had to be removed to allow correct access later for coronectomy sectioning (Fig. 2 b). The second and third molar areas were cut from the model in object mode by creating a plane and using the Boolean function. A splint was created to make them easily changeable to reduce the costs of 3D printing. It was done in edit mode by creating a T shape, extracting it from the second and third molar areas, and attaching it later to the base of the model. Minor bone removal was done virtually in sculpt mode (bone crest ~ 2 mm below the cemento-enamel junction) (Fig. 2 c). Afterwards, the redefined right side of the model with the changeable second and third molar areas was replicated to the other side in a mirror image by using the mirror modifier. This resulted in the desired experimental jaw model, in which the right and left third molars had the same impaction status. The base of the model was designed in edit mode from images acquired by 3D scanning (Artec Space Spider, Artec3D, Luxembourg) of a DRSK jaw model base (DRSK Restorative jaw, DRSK, Sweden) (Fig. 2 d) to fit the commercially available, standard phantom heads used in the department (G40 Jaw simulator with standard face mask, KaVo, Bieberach, Germany).

The appearance and structure of our above-described 3D-printed jaw model was based on the findings of Feng et al. [34]. For individualization, following requirements were considered in the current study: i) easily changeable distal molar segment (second and third molars together with retromolar area) to reduce costs; ii) mirror-symmetric design of the left and right distal “molar-segment”; and iii) maximally compatible regarding all dimensions and the connecting structure for the commercially available, standard phantom heads used in the department.

### Jaw-model fabrication

**Fig. 3:**
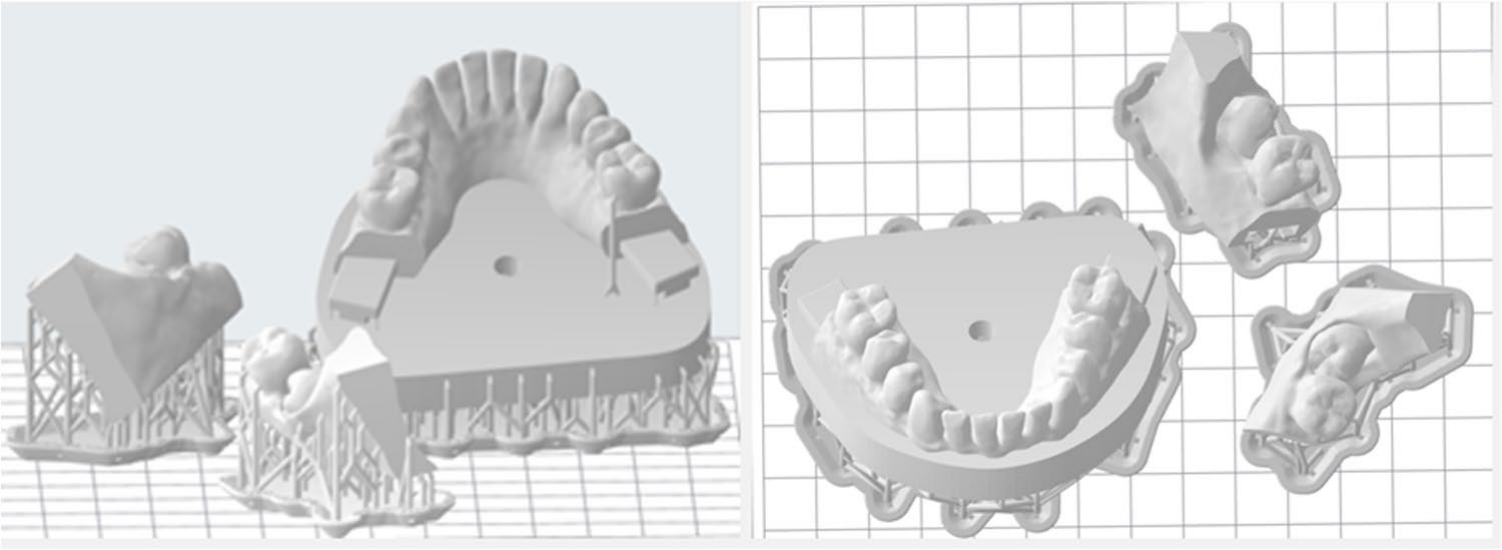
The jaw model in the PreForm software, illustrating the model orientation and support generation

The model was fabricated using a resin (White Resin, V4, Formlabs, Boston, MA, USA) suitable for additive manufacturing purposes. The selected material has a favorable characteristic in terms of imaging, and the CBCT scans of the model had excellent quality. Also, it is hard (Shore D hardness = 82) and drillable, which is important regarding the optimal haptic feedback of the fabricated tooth. The models were printed out using a standard desktop stereolithography (SLA) printer (Form 2, Formlabs, Boston, MA, USA), with 0.1 mm layer height. Setting the orientation on the printing bed and generating the support were done using the PreFrom software (Formlabs, Boston, MA, USA) (Fig. 3*.*).

**Fig. 4:**
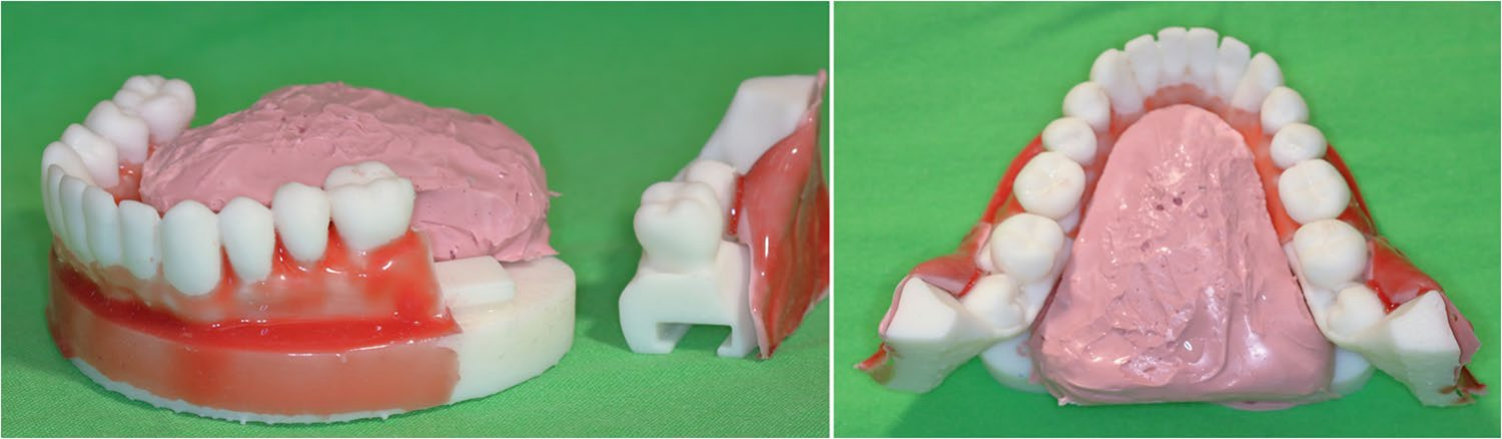
The printed jaw model with colored silicon “gingiva” coating and “tongue”. The change of retromolar segments was simple and fast, while the anatomy of the third molar area was entirely symmetric

The jaw model was fitted with red pigment-colored silicone (Rebound 25, Smooth-On, Texas, USA) sheet covering to mimic buccal soft tissues and to mimic a real mucoperiosteal flap (in combination with a first layer of silicone impression material), to necessitate flap raising and retraction during tooth sections. A tongue was also constructed from silicone impression material (ZA 22 Thixo Body, Zhermack, Badia Polesine, Italy) to cover and hide lingual surfaces similar to in vivo situations (Fig. 4).

### Surgical equipment and drill sleeve fabrication

**Fig. 5:**
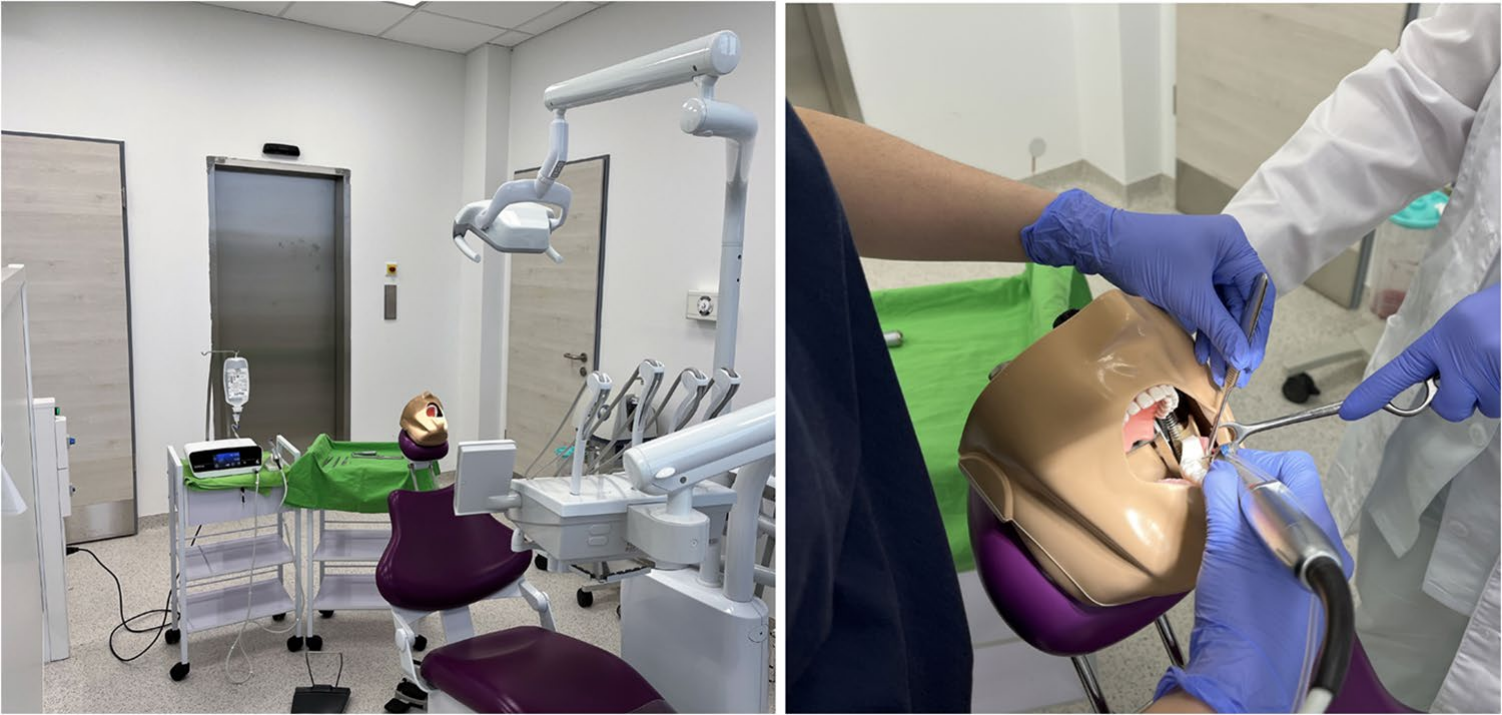
For simulation-based interventions, the same surgical setting was used as in clinical operations, and the same experienced nurse assisted during the procedures. Phantom heads were attached to the headrest

**Fig. 6:**
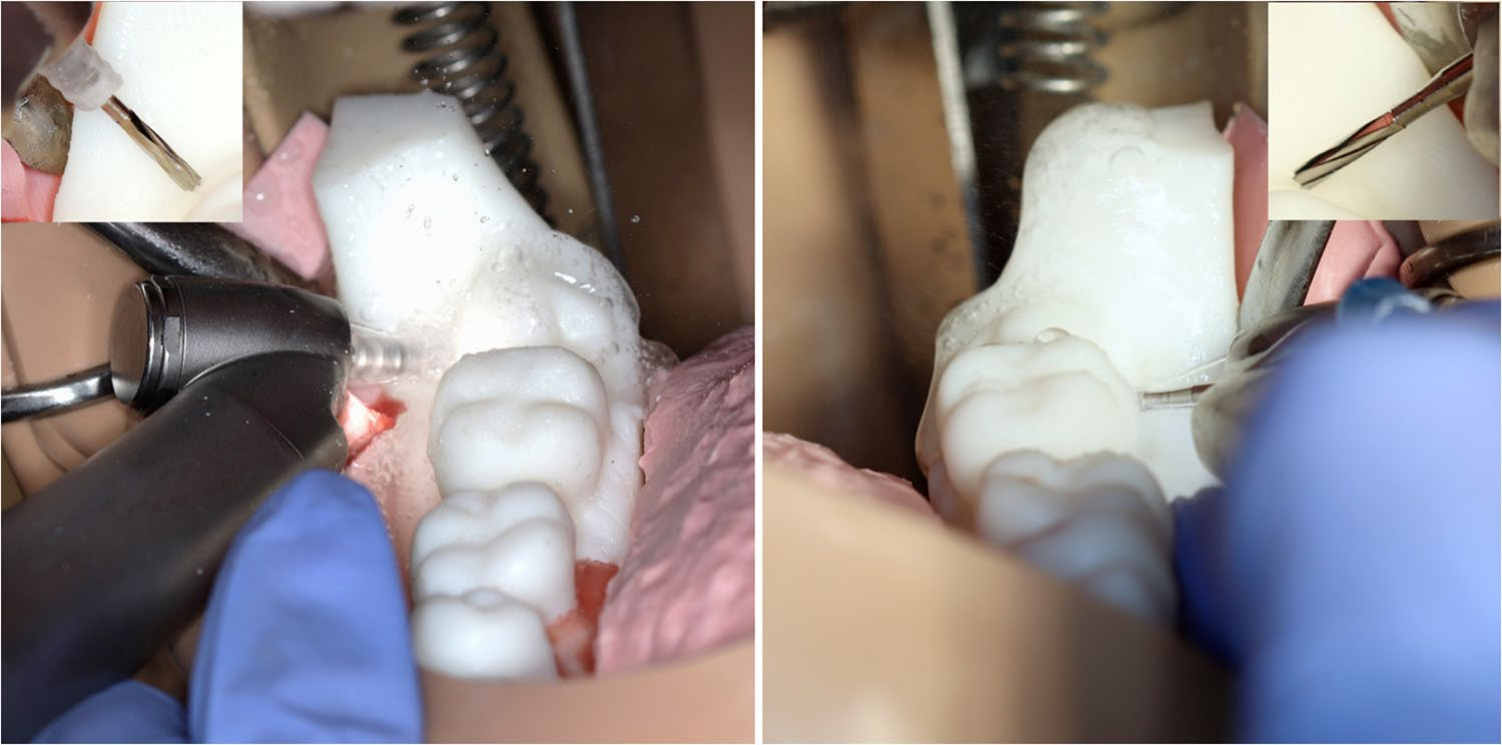
Coronectomy was performed on one side using a drill sleeve; it was performed freehand on the other side

The simulated surgery was intended to mimic real clinical situations (Fig. 5). The phantom-head was fixed in the headrest of a dental chair (Primus 1058 Life, KaVo, Bieberach, Germany) with double sided stick-tape (3 M Heavy Duty Molding Tape, 3 M Hungária Kft, Budapest, Hungary). A surgical physio-dispenser (MASTERsurg, KaVo, Bieberach, Germany) unit was coupled with a surgical 45° angulated accelerator handpiece (1:3 speed increasing ratio; TiMax Z-SG45L, NSK-Nakanishi, Eshborn, Germany). For crown sectioning, irrigation was set to 50 ml/min, and drilling speed was set to 120,000 revolutions per minute. Coronectomy cuts were performed with a tungsten carbide fissure drill (HM21L, Hager & Meisinger GmbH, Neuss, Germany). The applied 3D-printed drill sleeve (DS) (Fig. 6), including manufacturing and clinical usage, was introduced in detail in an earlier work [31]. The 3D CAD model of the sleeve was designed using AutoDesk Inventor® (AutoDesk, San Francisco, CA, USA), based on the measured drill’s values. The sleeve was printed using a Stratasys PolyJet™ J750 (Stratasys Ltd. Eden Prairie, Minnesota, USA) 3D printer. The sleeve’s material was a mixture of UV-hardened photopolymer (Stratasys MED670 VeroDent™), with the layer height („Z” resolution) of 16 µm. This material is approved and extensively employed in medicine and dentistry. The printed sleeve was then fixed by friction on the shaft of the above-mentioned drill. Additionally, drill selection was based on the results of another investigation [26]. Participants were informed about the length of the sleeves and that it would leave 1 mm tooth material lingually intact when the drill was applied as deep as the sleeve allowed.

### Study groups

Thirty-six colleagues of our department either in dentistry or in oral or maxillofacial surgery training or as specialists were involved in this experiment. All the colleagues were monitored before selection (by evaluating the annual medical records of the department) regarding the number of surgeries involving lower impacted third molars and coronectomies performed by them. Less experienced colleagues were mainly trainees, with more than 30 but less than 100 impacted third molar removals and more than 3 but less than 10 coronectomies during their careers. Experienced colleagues were mainly specialists or trainees just before specialization, with a minimum of 500 impacted third molar removals and 50 coronectomies in the last 5 years.

All the included colleagues had to perform coronectomies bilaterally, on one side with DS (∑n = 36, DS) and on the other side without it (∑n = 36, FH) (Fig. 6). This resulted in a total of 72 coronectomies. Both starting side (left or right) and the used method on the current sides (DS or FH) were randomly selected by tossing a coin.

Before the surgery, candidates had the possibility to analyze the anonymized CBCT of the patient. For the analysis, the “InvivoViewer” software (version 2.0.0., KAVO) was used with a desktop computer (32 in, 2560 × 1440 resolution, Quad HD (QHD) monitor, Q32P2, AOC, Taipei, Taiwan).

### Collection and evaluation of data

**Fig. 7:**
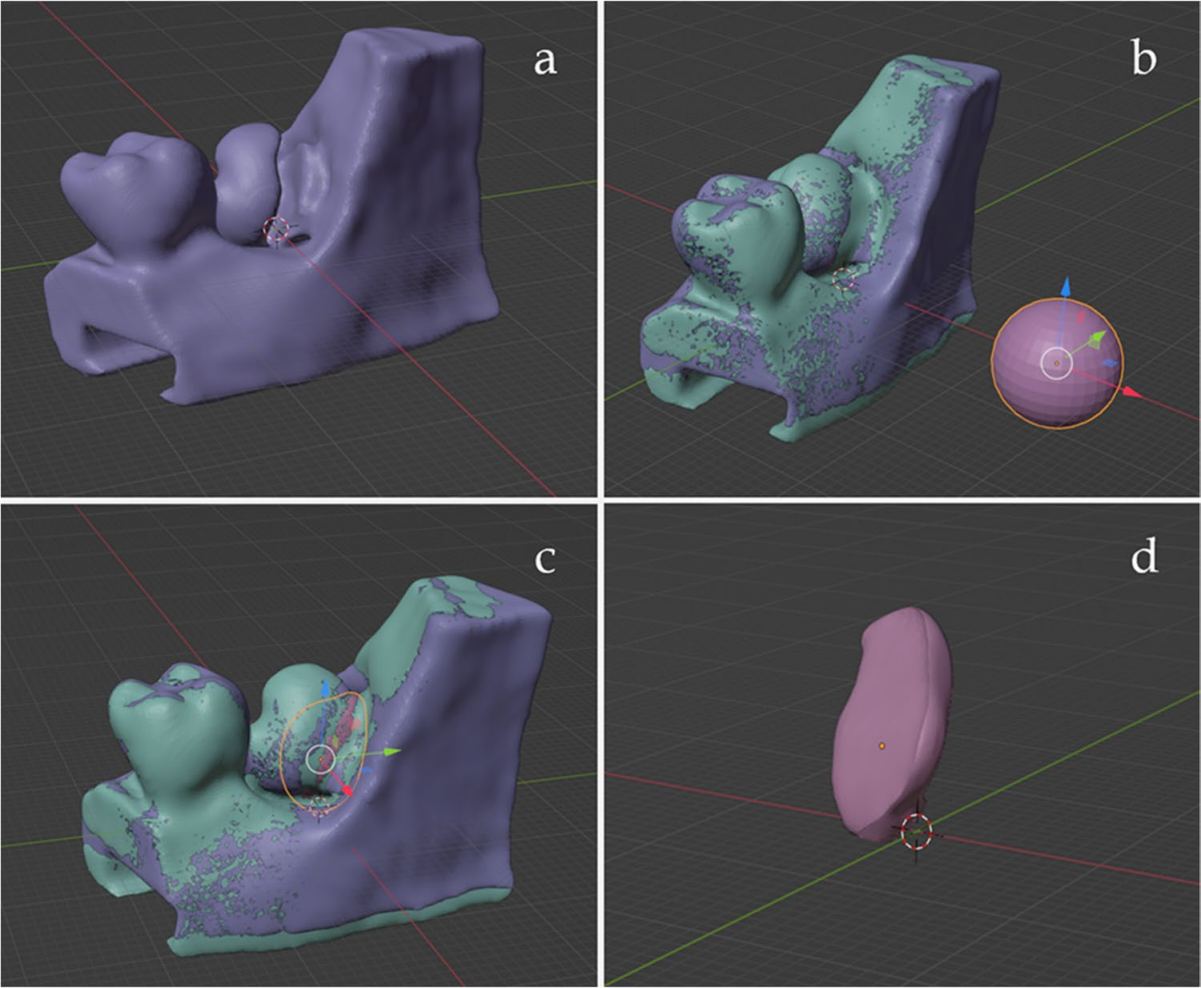
**a** The virtual model of the scanned left-side retromolar segment after coronectomy. **b** The comparison of the drilled segment with the intact segment and the creation of a sphere. **c** The sphere was precisely formed to fill the drilled area. Preoperative and postoperative segments were transformed into the same coordinate system. **d** The dimensions of the cut after using the Boolean function

During the interventions, the operation time was registered with a stopwatch. After the interventions, the retromolar segments were annotated with an identification number. Then, the segments were scanned with the abovementioned CBCT device. The DICOM files in.dcm format obtained from the CBTC scans were imported to the 3D Slicer software, and the segmentation was done at the 1.78–489 threshold. Irrelevant structures were removed, and the models were exported as.stl files to the Blender software (Fig. 7). In the software, the drilled retromolar segments were compared with the intact retromolar segments. A sphere was created, that was precisely formed to fill the drilled area of the teeth. Then, the Boolean function was used to obtain the dimensions of the cut.

**Fig. 8:**
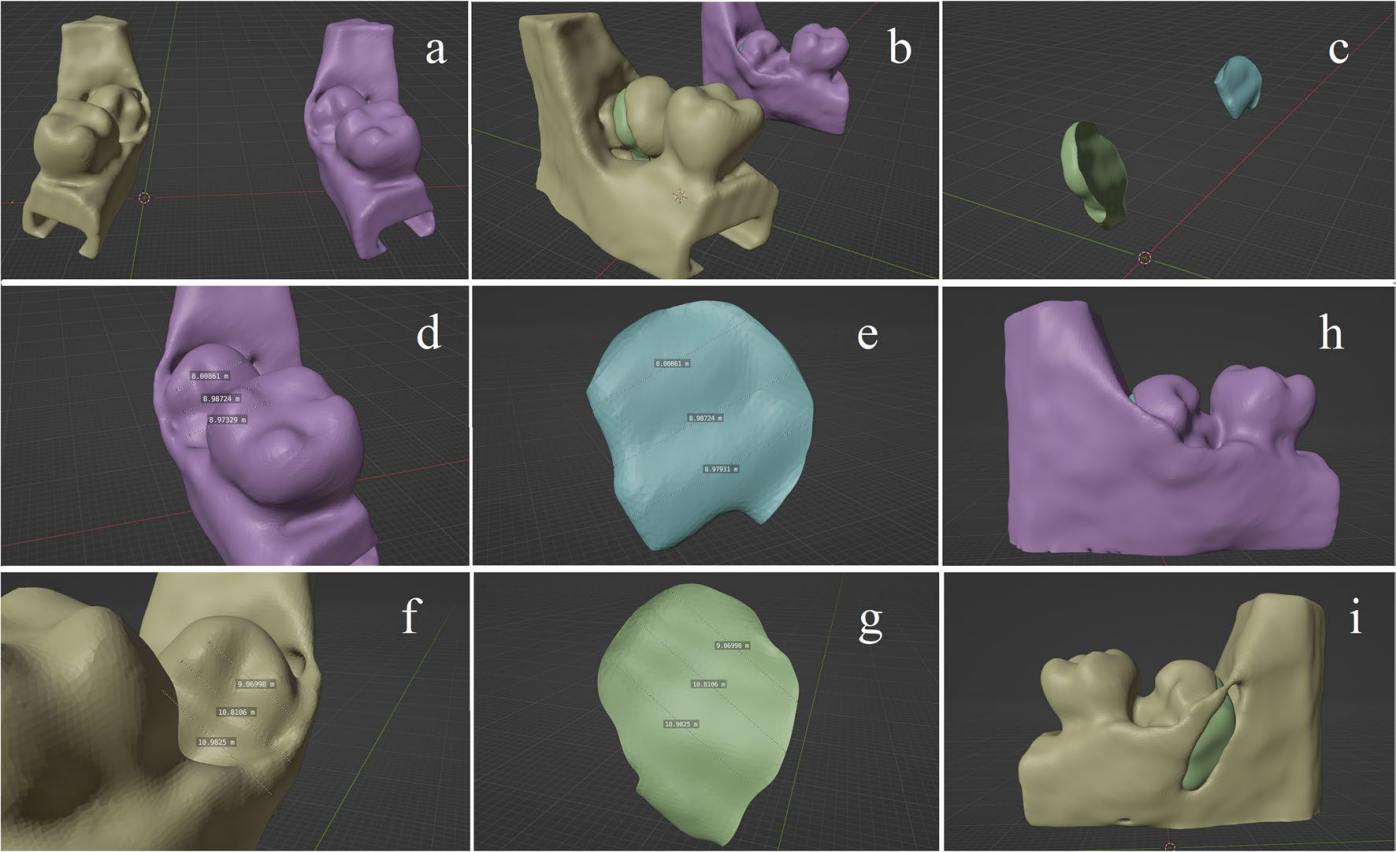
**a** The virtual model of the scanned retromolar segments after coronectomy. **b**, **c** The subtracted coronectomy sections could be examined from all directions precisely. **d**, **e** The right (freehand). **f**, **g** The left side (drilling sleeve) coronectomies of the same surgeon in this investigation with depth measurement values. **h**, **i** The same coronectomies from a lingual perspective. With the drilling sleeve (**h**), the lingual cortical was intact; however in the freehand side, (**i**), a significant defect was visible across the lingual cortex

In the Blender software, it was possible to analyze precisely the exact coronectomy cut in three dimensions (Fig. 8). The buccolingual cutting depth was analyzed at three standard locations, that is, at the vertical sections of the mesial and distal cusps and at the vertical section along the lateral fissure. These measurements resulted in a mesial, a middle, and a distal cutting length value of each coronectomy sections. Two of the authors measured all the cutting depths and the means of their measurement values were used. In addition, the intra- and inter-observer reliability of these two authors was calculated.

To establish an optimal cutting depth, a reference (zero) point was created in the horizontal cut section virtually by 1 mm distance of the lingual surface of the third molar tooth in the three abovementioned buccolingual sections/plans. When cuts were deeper (= longer) by ≥ 2.0 mm as the reference point, cuts were judged as “too deep” as the lingual structures were prepared ≥ 1 mm deeper than the tooth. In case of shorter drillings, a “too superficial” buccolingual cut decision was made, when the cut was shorter by ≥ 3 mm than the reference point. In these cases, ≥ 4 mm of the tooth material remained unprepared lingually, which was approximately ≥ 40% of the buccolingual dimension of the tooth.

### Statistical analysis

For statistical analysis, the SPSS version 26.0 (IBM Corp., Armonk, NY, USA) and MedCalc (Ostend, Belgium) statistical software were used.

The required sample size was calculated based on the data from a previous pilot drilling series, including 10 FH and 10 DS coronectomies, according to Padam (2012) [35]. Mean outcomes were assumed 7.6 mm (FH group) and 8.8 mm (DS group), and standard deviations were assumed 2.0 mm (FH group) and 1.0 mm (DS group). Under an alpha threshold of 0.05 and a power of 80%, a sample size of 36 per group was estimated.

The associations of “too short” and “too deep” cuts with the applied techniques (FH vs. DS) or between groups (experienced vs. inexperienced) were tested by Fisher’s exact or Chi-squared tests and odds ratios were calculated. The deviation between the virtually planned and realized cutting depths were compared using the independent samples t-test. To compare the drilling times of the two sides or between groups, or both, the Mann–Whitney *U* test was used. A *p*-value less than or equal to 0.05 was considered statistically significant.

Intra- and inter-observer reliability were calculated by Cohen’s kappa test, after both observers analyzed 20 images one week later than the first round of analysis. A kappa value of < 0.40 was considered to show poor agreement, a value of 0.40–0.59 fair agreement, a value of 0.60–0.74 good agreement, and a value of 0.75–1.00 excellent agreement.

## Results

Regarding cutting depths, in 7 cases, the tooth sections were “too deep” in the FH group, while none of the sections went through the entire tooth surface in the DS group. Without the drilling sleeve, there was an odds ratio (OR) of 18.56 [95% confidence interval (95% CI): 1.02–338.5; *p* = 0.048] for the occurrence of a “too deep” tooth section. A “too superficial” buccolingual section occurred in 18 cases of the FH group, while in only 8 cases in the DS group (OR: 3.50; 95% CI: 1.26–9.72; *p* = 0.016) (Table 1).

**Table 1:** The occurrence of suboptimal sections in the different sectioning technique groups

Suboptimal sections	Sectioning technique		OR	95% CI		P value
	Freehand	Drill sleeve		Lower	Upper	
Deep (n)	7	0	18.56	1.02	338.5	0.048
Superficial (n)	18	8	3.50	1.26	9.72	0.016

*OR* odds ratio, *CI* confidence interval

The deviation between virtually planned (i.e., optimal) and realized cut depths was significantly greater in the FH group (1.91 ± 1.62 mm) than in DS group (1.21 ± 0.72 mm) (p < 0.001; t-value: -4.764; independent samples t-test).

Of the 36 participating colleagues, 20 were classified in the less experienced, while 16 in the experienced group. The occurrence of “too deep” FH sections were similar in the experienced (3 cases) and in the less experienced (4 cases) groups (*p* = 0.983; Fisher’s exact test). With the FH technique also, the occurrence of “too superficial” cuts were similar in the 2 groups (11/20 vs. 7/16; *p* = 0.697; Chi-squared test). Of these 18 “too short” cut FH cases, the shortest drilling values were most frequently seen distally, along the distal roots/cusps (OR: 6.76; 95% CI: 1.57–29.07; *p* = 0.01) (Table 2).

**Table 2:** The occurrence of suboptimal sections related to the level of experience with different methods

Sectioning technique	Suboptimal sections	Level of experience		*p*-value
		Inexperienced	Experienced	
Freehand	Deep (n)	4	3	0.983*
	Superficial (n)	11	7	0.697**
Drill sleeve	Deep (n)	0	0	Not calculated
	Superficial (n)	5	3	0.655*

* Fisher’s exact test, ** Chi-square test

Regarding the duration of the operation, it was obvious that FH coronectomies (119.93 ± 106.50 s) were longer in duration than the DS coronectomies (82.27 ± 80.69 s), which was significant (*p* = 0.021; Mann–Whitney *U* test). However, when the operation time differences were evaluated separately in the experienced and less experienced groups, a significant difference was observed only in the less experienced group (FH: 158.95 ± 125.61 s; DS: 106.92 ± 100.79 s) (*p* = 0.038; Mann–Whitney *U* test). In addition, experienced colleagues (65.31 ± 26.54 s) were significantly faster with FH than less experienced colleagues (158.95 ± 125.61 s) (*p* < 0.001; Mann–Whitney *U* test). Moreover, experienced colleagues (79.08 ± 16.98) were also faster when using the drilling sleeve compared with the unexperienced group (106.92 ± 100.79 s) (p = 0.004; Mann–Whitney *U* test).

The two investigators had excellent (0.97 and 0.95) intra-observer and excellent (0.91) inter-observer reliability based on the kappa values in this study.

## Discussion

The accuracy of tooth sections is an important determining factor for successful coronectomy procedures. This study proved that the precision of the buccolingual cutting depth can be improved, while surgical time can be reduced when applying a drilling sleeve.

A direct contact between the LN and the third molar alveolar wall can occur in up to 62% of the cases, and in up to 17.6% of the cases the LN can be located at the same level or above the top of the ridge [28]. During third molar tooth sectioning, LN can be involved by the bur. Moreover, lingual cortical fenestrations—both preexisting anatomic and iatrogenic—may be significant predisposing factors in nerve injury [28, 36]. Therefore, a subtotal section and completing it with nonrotating hand instruments can be recommended [9, 22, 28, 31, 36, 37]. In contrast, some authors recommended rather complete crown sectioning to reduce the possibility of root mobilizations [20, 23, 38]. In case of complete crown sections, however, lingual tissues require protection. Lingual flap retraction is an alternative step in trying to reduce lingual soft tissue’s injury [27]. However, a systematic review found that LN injury is 8.8 times higher in case of a lingual flap retraction [39]. Another meta-analysis showed this odds ratio to be 4.8 (lingual flap retraction vs. no retraction) and concluded that the cumulative prevalence of permanent LN injury was found to be 0.07 ± 0.21% with lingual flap retraction, 0.18 ± 0.38% without it, and 0.28 ± 0.48% using the lingual split technique [40]. Moreover, Pippi et al. stated that it seems preferable to avoid lingual flap elevation [28]. From the abovementioned points of view, increasing the precision of buccolingual splits can be primarily supported. Without the drilling sleeve, there was ~ 18.6 times higher risk for drilling minimum 1 mm deeper as the lingual tooth contour, while ~ 3.5 times higher risk for leaving minimum 4 mm of the tooth material unprepared lingually. The thickness of the remaining lingual tooth material after sectioning can highly determine the force needed for crown fracturing. According to Xu et al., the breaking force of the crown by 3 mm residual tooth tissue is 3.46 times higher, while by 2 mm residual tooth tissue 3 times higher than forces by 1 mm remaining tooth material (70.20 vs. 60.99 vs. 20.13 N) [41]. Thicker residual tooth tissue results in higher breaking forces, which may lead to more frequent root mobilizations. Additionally, the odds for this was ~ 6.8 for a short suboptimal section, located by the distal root. That attitude might also be a result of the earlier learned or experienced fear regarding the vulnerability of the distolingual area of the third molar. Despite using the drill sleeve, in some cases, a “too short” section depth occurred. According to feedback from colleagues involved, this was due to the fear of decreasing tooth diameter mesially or distally because of the usual elliptical cross-section of the third molar tooth, that is, the convex buccal tooth surface. With intention, they tried to compensate for this mild convexity but with little success. After unmasking the results, it became obvious that the drill sleeve’s diameter was large enough relative to the surface convexity to compensate for it. Based on this feedback, a drill sleeve may require a short learning curve.

The mean lingual bone thickness was found to be 1.21 ± 0.63 mm at the cemento-enamel junction of the second molar [42], while the thinnest bone at third molars was 0.78 ± 1.27 mm in Menziletoglu et al.’s study and 0.55 ± 0.48 mm in Momin et al.’s study [42, 43]. Furthermore, in ~ 20.5% of the patients the lingual plate was found to be fenestrated at the middle-third of the root of the third molar [44]. Thin or fenestrated lingual alveolar wall was found to be correlated with higher incidence of LN injury [45, 46]. The thinnest lingual plates were seen in the case of horizontal and distoangular angulations [47] or in case of horizontal and mesionagular angulations [44]. Another study confirmed that as the buccolingual or mesiodistal angulations increase, lingual bone thickness decreases [42]. In the case used for this study, the thinnest lingual bone thickness was 1.15 mm at the CEJ, in the cross-sectional CBCT slides.

Coronectomy procedures require a specific learning curve [48]. In the study by Monaco et al., it was found that surgeons with greater expertise (≥ 10 years) could statistically lower the incidence of complications [48]. Less experienced surgeons took longer to complete the procedure, and, for example, postoperative pain was correlated with the duration of surgery [48]. Although the experience of the operating surgeons was not determined exactly in that study, it seems obvious that less experienced colleagues should be supported in all cost-effective and reasonable ways during coronectomy procedures. In the current study, the coronectomy durations were significantly shorter with drilling sleeve when performed by inexperienced colleagues. Sectioning times were reduced to ~ 67% compared to FH sections. In other words, inexperienced trainees were ~ 2.44 times slower using FH but only ~ 1.35 times slower with DS when compared with experienced surgeons. In contrast, Zeng et al. found that drillings with their third molar surgical splitting guide were significantly slower—both in experienced and inexperienced groups—compared to tooth sections made freehand [33]. Additionally, in our opinion, the drilling sleeve causes less disturbance in visibility and in the flow of the irrigation liquid than a splitting guide.

Based on earlier clinical testing experience, for DS coronectomies, all procedures were performed with a new sleeve. During the current experiment, it became obvious that in cases where the sleeve was pressed too strongly on the tooth surface, the sleeve showed signs of wear. This wear manifested itself first in a rounded apex and then in a further decreasing diameter at the tip, resulting in a conical shape. It seems that drill sleeves should be used only once.

Another aspect is cost effectiveness and availability for clinicians. On the one hand, sleeve printing is very cost-effective, less than 3 €/piece of pure material cost. However, the prices of sleeve design and 3D printing services should also be considered. In our opinion, this is very similar to any known guided surgical procedure (implantation, periapical surgery, tooth transplantation or endodontic trepanation), where the clinician needs access to dedicated software to design the sleeve/guide/and subsequently print the sleeve. However, it should be mentioned that the drill sleeve can be adjusted with a scalpel. It is possible to print several pieces, such as 1 cm sleeves optimizing costs, and after that, the sleeves can be adjusted to the appropriate size for the current clinical case [31].

Based on our clinical experience, some impaction patterns and clinical situations (e.g., decreased mouth opening and scleroderma) that can limit the admittance of the examined handpiece may also limit the use of the drill sleeve. Regarding impaction status, in deep (Pell & Gregory [P&G]: B or C type) horizontally angulated, or in case of deep (P&G: B or C) pronounced mesio-, and distoangular impactions, using a sleeve can be complicated or impossible.

This study had some limitations. For the coronectomies a 3D-printed jaw model was used. Training models are not able to entirely simulate real clinical patients. The model was not bleeding, did not produce any saliva, neither the tongue nor the patient was moving during the procedure, and did not give any reactions or responses to pain. As opposed to this, the 3D-printed resin teeth gave slightly different haptic feedback during drilling; however, according to the participating experienced surgeons, drilling the 3D-printed teeth was not significantly different from drilling a real tooth. This agreed with an earlier study, where a similar model received very positive feedback from the participating oral surgeons [34]. To support similarities between 3D-printed teeth and real human molars, an earlier study could be mentioned using the same handpiece and tungsten-carbide drill for in vitro coronectomy sections [26]. The average preparation times by experts in the current study (~ 65 s) were entirely comparable with drilling times necessary to section extracted fresh human molars (between ~ 53–61 s).

It is also important to note that the optimal drilling depth in this study, leaving 1 mm of intact lingual tooth tissue, was determined based on our experiences. There is no consensus data or international guidelines determining the optimal sectioning depth. However, suboptimal drilling depths applied in this study can bear clinically significant risks. In the case of “too deep” sections, considering the average human lingual bone thickness, lingual soft tissues can be prepared. In the case of “too superficial” sections, however, more than 40% of the crown remains unprepared. In such cases, crown fracturing can be highly unpredictable, based on our experience, and total tooth luxation can occur.

Despite these drawbacks, only this method was able to offer an identical third molar anatomical situation for every single coronectomy performed in this study.

## Conclusions

The drill sleeve was found to be an effective tool in reducing operation times by less experienced colleagues during coronectomy of tooth sections. Moreover, irrespective of the level of experience of the participants, it avoided any drilling outside of the lingual tooth contour. In addition, the drill sleeve was also helpful in avoiding “too superficial” suboptimal cuts by leaving significantly thinner tooth crown material lingually intact.

### Supplementary Information

Below is the link to the electronic supplementary material.

Supplementary file1 The cone-beam computed tomography images of the patient which was used for model construction in this study. In the axial slice, tooth dimension and lingual bone thickness are shown. (TIF 34098 KB)

## Data Availability

The datasets used and/or analyzed during the current study are available from the corresponding author upon reasonable request.
