# Supplementary figures and images for: The effect of individual drilling sleeves on the precision of coronectomy tooth sections. An in vitro 3D-printed jaw model experiment
Source: Clin Oral Investig. 2023 Oct 3;27(11):6769–80. doi: 10.1007/s00784-023-05289-4 (PMC10630220; doi:10.1007/s00784-023-05289-4)

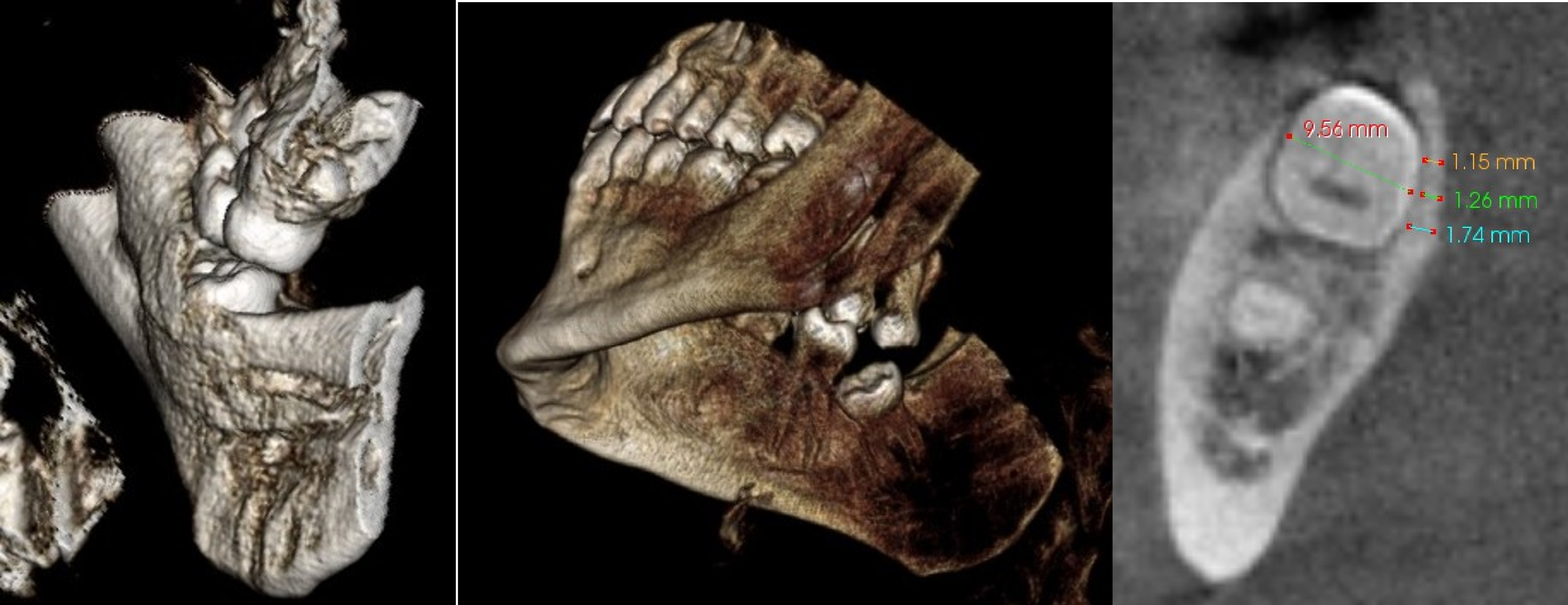

Supplement: Supplementary file 1 — Supplementary file1 The cone-beam computed tomography images of the patient which was used for model construction in this study. In the axial slice, tooth dimension and lingual bone thickness are shown. (TIF 34098 KB) [file 784_2023_5289_MOESM1_ESM.tif]
